# Caveolin-1 Regulates Endothelial Adhesion of Lung Cancer Cells via Reactive Oxygen Species-Dependent Mechanism

**DOI:** 10.1371/journal.pone.0057466

**Published:** 2013-02-27

**Authors:** Pithi Chanvorachote, Preedakorn Chunhacha

**Affiliations:** Department of Pharmacology and Physiology, Faculty of Pharmaceutical Sciences, and Cell-based Drug and Health Product Development Research Unit, Chulalongkorn University, Bangkok, Thailand; China Medical University, Taiwan

## Abstract

The knowledge regarding the role of caveolin-1 (Cav-1) protein on endothelium adhesion of cancer cells is unclear. The present study revealed that Cav-1 plays a negative regulatory role on cancer-endothelium interaction. Endogenous Cav-1 was shown to down-regulate during cell detachment and the level of such a protein was conversely associated with tumor-endothelial adhesion. Furthermore, the ectopic overexpression of Cav-1 attenuated the ability of the cancer cells to adhere to endothelium while shRNA-mediated Cav-1 knock-down exhibited the opposite effect. We found that cell detachment increased cellular hydrogen peroxide and hydroxyl radical generation and such reactive oxygen species (ROS) were responsible for the increasing interaction between cancer cells and endothelial cells through vascular endothelial cell adhesion molecule-1 (VCAM-1). Importantly, Cav-1 was shown to suppress hydrogen peroxide and hydroxyl radical formation by sustaining the level of activated Akt which was critical for the role of Cav-1 in attenuating the cell adhesion. Together, the present study revealed the novel role of Cav-1 and underlying mechanism on tumor adhesion which explain and highlight an important role of Cav-1 on lung cancer cell metastasis.

## Introduction

Recently, roles of caveolin-1 (Cav-1) in regulation of cancer progression and metastasis in various types of cancer have been revealed [1]–[4] and such a protein perhaps received the most attention in cancer-related research. Although some studies suggested that Cav-1 may play a role in inhibiting cancer progression in certain cancers [5], in lung cancer, Cav-1 potentiates cancer aggressiveness as well as metastasis [6]. Together with the fact that Cav-1 expression in lung cancer was shown to relate to poor prognosis [2], and most of the cancer-related death in this cancer was shown to link with metastasis, it is of great interest to investigate the entire regulatory role of this protein on cancer metastasis [7]. Metastasis is a multi-step process of cancer cells spreading from their original locations to the distant secondary sites. Starting with the cancer cell detachment from their primary tumor, the cells invade vascular wall, travel in the circulatory system, and adhere to the endothelium to form the secondary tumors. Although roles of Cav-1 on lung cancer cell behaviors have been intensively explored, the role of such a protein on lung cancer cell adhesion to endothelium surface is largely unknown. We and others have suggested the important role of Cav-1 in rendering cancer cells resistant to anoikis after cell detachment [6], [8], [9], [10], enhancing invasion and migration [11], and facilitating growth in anchorage-independent manner [12]. Endogenous Cav-1 level was shown in the previous studies to be controlled by the reactive oxygen species (ROS). In detached cell condition, hydrogen peroxide was shown to increase the cellular level of Cav-1 by inhibiting its degradation [6]. While in the adherent cells, hydroxyl radical was shown to be a key player in up-regulating Cav-1 expression and increased cell migration [11]. These findings highlighted the regulatory role of ROS on Cav-1 expression and their accompany roles on cancer metastasis.

In biology, negative feedback regulations exist to prevent the excessive stimulations. Likewise, Cav-1 protein was shown to suppress oxidative stress caused by hydrogen peroxide exposures [13]. However, it remains unknown whether Cav-1 regulates ROS level in detached cells and such regulation is critical for cancer adhesive property. Using pharmacological and genetic approaches, the present study revealed that Cav-1 plays a key role in inhibition of cancer-endothelium adhesion by attenuating hydrogen peroxide and hydroxyl radical generations after cell detachment. The present study also found that Cav-1 suppressed such ROS formation through Akt-dependent mechanism. Along with the observation that Cav-1 decreased in a time-dependent fashion after cell detachment, we found that at later-time points, cancer-endothelium adhesion significantly increased the concomitant of that Cav-1 depletion. Thus, our study revealed the existence of a novel mechanism of cancer cell adhesion regarding Cav-1 which might be exploited in metastasis and drug design.

## Materials and Methods

### Cells and Reagents

Non small lung cancer cell (NSCLC)-H460 and Vascular endothelium Human (HUV-EC-C) cells were obtained from the American Type Culture Collection (Manassas, VA). H460 cells were cultured in RPMI 1640 while HUV-EC-C cells were cultured in M199 medium. RPMI 1640 was supplemented with 5% fetal bovine serum (FBS), 2 mM L-glutamine, and 100 units/mL penicillin/streptomycin. M199 was supplemented with 10% fetal bovine serum (FBS), 10 mM L-glutamine, and 100 units/mL penicillin/streptomycin, 0.1 mg/ml heparin, 0.05 mg/ml endothelial cell growth supplement (ECGS). All of the culture was incubated in a 5% CO_2_ environment at 37°C. 2′, 7′-dichlorofluorescein diacetate (DCFH_2_-DA), Dimethysulfoxide (DMSO), caveolae isolation kit, Calcein AM, Heparin sodium were obtained from Sigma Chemical, Inc. (St. Louis, MO); Rabbit caveolin-1 antibody and peroxidase-conjugated secondary antibody from Abcam (Cambridge, MA); Hydrophenyl fluorescein (HPF), LY294002, Amplex Red, Lipofectamine 2000 were from Invitrogen (Carlsbad, CA); Antibody for β-actin from Santa Cruz Biotechnology (Santa Cruz, CA); Antibody for pan-Akt, p473-Akt, PTEN, EGFR, Phospho-PTEN (Ser380/Thr382/383) were from Cell Signaling Technology, Inc. (Danvers, MA); Endothelial cell growth supplement was from Millipore Corporation (Billerica, MA).

### Plasmid and Transfection

The Cav-1 expression plasmid pEX_Cav-1 and its control vector; pDS_XB-YFP were acquired from the American Type Culture Collection (Manassas, VA) and Cav-1 knockdown plasmid shRNA-Cav-1 and its control vector; control shRNA plasmid A were obtained from Santa Cruz Biotechnology (Santa Cruz, CA). Stable transfections of Cav-1 expression plasmid or Cav-1 knockdown plasmid were generated by culturing H460 cells in a 6-well plate until they reached 60% confluence. 15 µl of Lipofectamine reagent and 2 µg of Cav-1, and shRNA-Cav-1 plasmid were used to transfect the cells in the absence of serum. After 12 h the medium was replaced with culture medium containing 5% fetal bovine serum. Approximately 36 h after the beginning of transfection, the cells were digested with 0.03% trypsin, and the cell suspensions were plated onto 75-ml culture flasks and cultured for 24 to 28 days with G418 or puromycin for selection of Hcav-1 and shCav-1, respectively. The stable transfectants were pooled and the expression of Cav-1 protein in the transfectants was confirmed by western blotting. The cells were cultured in antibiotic free RPMI 1640 medium for at least two passages before used in each experiment.

### ROS Detection

Intracellular ROS were determined by flow cytometry using DCFH_2_-DA as a fluorescent probe. Briefly, cells were incubated with 10 µM DCFH-DA, HPF for 30 min at 37°C, after which they were washed, trypsinized, resuspended in phosphate-buffered saline, and immediately analyzed for fluorescence intensity by FACScan flow cytometer (Beckton Dickinson, Rutherford, NJ) using a 488-nm excitation beam and a 538-nm band-pass filter. Median fluorescence intensity was quantified by CellQuest software (Becton Dickinson) analysis of the recorded histograms.

H_2_O_2_ was determined by Amplex Red reagent (Invitrogen). Briefly, the reaction mixture containing 50 µM Amplex Red reagent and 0.1 U/ml HRP in Krebs-Ringer phosphate (KRPG) was added into each microplate well. Subsequently, 20 µl of 1.5×10^4^ H460 cells suspended in KRPG was added to the reaction mixture. After 30 min incubation, the fluorescence signal obtained from the fluorescence microplate reader equipped for excitation in the range of 530–560 nm and emission detection at 590 nm was monitored and measured over the 3 h post-detachment.

### Monolayer Cell Adhesion Assay

HUV-EC-C were stimulated with 10 ng/ml IL-1*β* for 0–4 h. H460 cells (2.5×10^4^ cells/ml) were stained with 15 µM of calcein AM (Sigma) for 45 min at 37°C and 5% CO_2_, then added onto a semi-confluent monolayer culture of HUV-EC-C, incubated for 20 min at 37°C with rotation at 120 rpm, and washed extensively to exclude nonspecific cell attachment. The number of attached cells was counted directly under a fluorescence microscope as reported previously [14]. For antibody mediated blocking of cell adhesion, the IL-1β –stimulated HUV-EC-C were incubated with antibody to VCAM-1, ICAM-1 and E-selectin (at a final dilution of 1∶400 for every antibodies) for 3 h at 37°C in humidified CO2 incubator, and then H460 cells were added.

### Caveolae Isolation

Caveolae were isolated from shCav-1, H460 and Hcav-1 cells by using a caveolae isolation kit (Sigma). ShCav-1, H460 and Hcav-1 cells were collected and lysed with lysis buffer containing 1% Triton X-100. The lysates were centrifuged at 10,000 *g* for 15 min. Detergent-resistant membrane fractions were isolated on OptiPrep Density Gradient Medium (0, 20, 25, 30, and 35%). Caveolae were collected from caveolin-1-enriched fractions, which has been used for the investigation of Cav-1 and PTEN expression.

### Western Blotting

After specific treatments, cells were incubated in lysis buffer containing 20 mM Tris-HCl (pH 7.5), 1% Triton X-100, 150 mM sodium chloride, 10% glycerol, 1 mM sodium orthovanadate, 50 mM sodium fluoride, 100 mM phenylmethylsulfonyl fluoride, and a commercial protease inhibitor cocktail (Roche Molecular Biochemicals) for 30 min on ice. Cell lysates were collected and determined for protein content using the Bradford method (Bio-Rad Laboratories, Hercules, CA). Equal amount of proteins of each sample (40 µg) were denatured by heating at 95°c for 5 min with Laemmli loading buffer, and subsequently loaded on 10% SDS-polyacrylamide gel electrophoresis. After separation, proteins were transferred onto 0.45 µm nitrocellulose membranes (Bio-Rad). The transferred membranes were blocked for 1 hour in 5% nonfat dry milk in TBST (25 mM Tris-HCl (pH 7.5), 125 mM NaCl, 0.05% Tween 20) and incubated with the appropriate primary antibodies at 4°C overnight. Membranes were washed twice with TBST for 10 min and incubated with horseradish peroxidase–coupled isotype-specific secondary antibodies for 1 hour at room temperature. The immune complexes were detected by enhanced with chemiluminescence substrate (Supersignal West Pico; Pierce) and quantified using analyst/PC densitometry software (Bio-Rad).

### Statistical Analysis

Mean data from independent experiments were normalized to result from cells in the control. All the experiments were repeated at least three times. A statistical analysis between two groups was verified by Student’s t test, in comparison to multiple groups, an analysis of variant (ANOVA) with post hoc test was conducted. The strength of relationships, correlation coefficient (*r*), between each protein level after detachment was determined with SPSS version 16 (SPSS Inc., Chicago, IL, USA). A P-value of less than 0.05 would be considered as statistically significant.

## Results

### Caveolin-1 Depletion after Cell Detachment Enhances Tumor-endothelial Cell Adhesion

**Figure 1:**
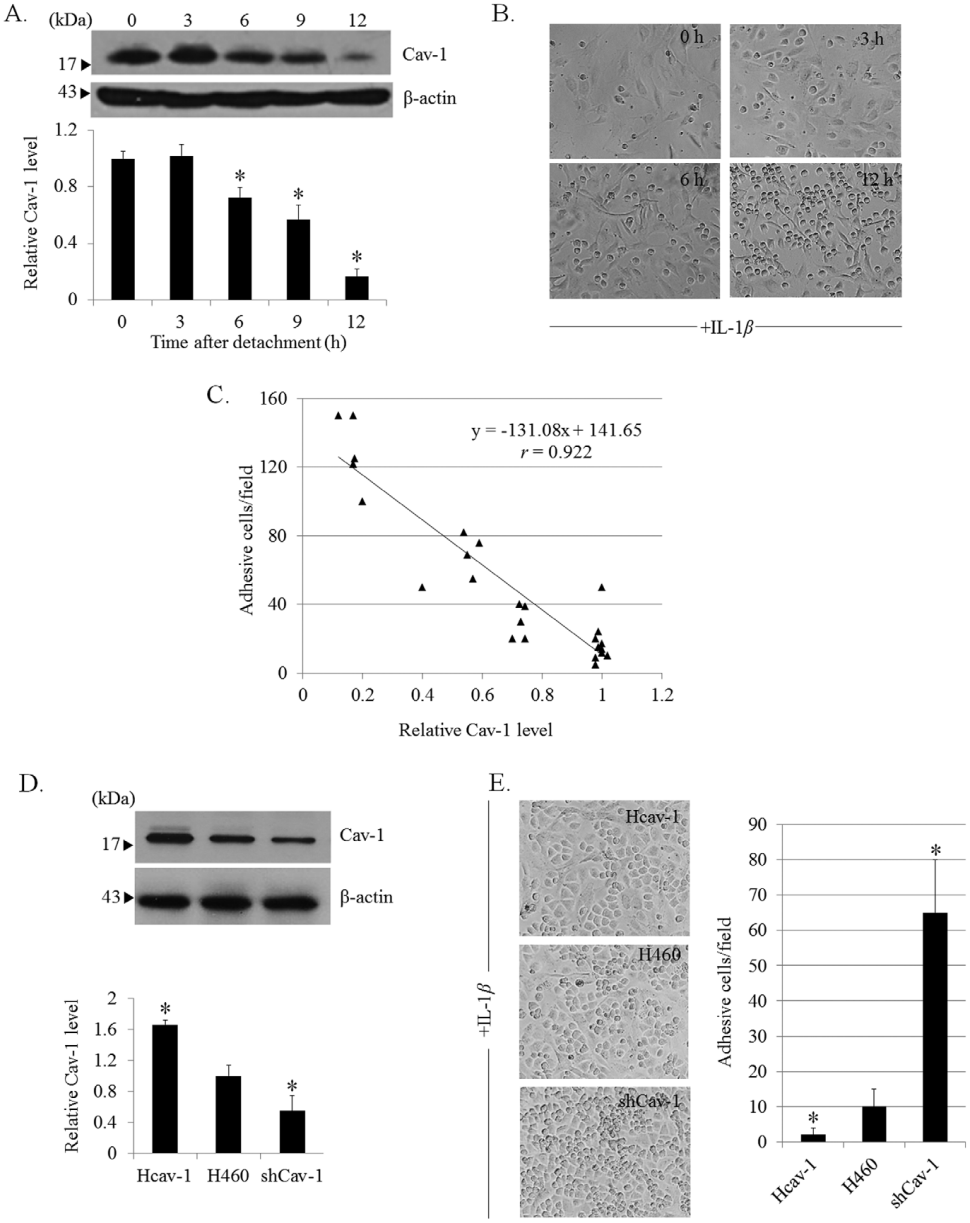
Caveolin-1inhibits cancer cell adhesion to HUV-EC-C endothelial cells. *A:* H460 cells were suspended in poly-HEMA-coated plates for various times (0–12 h) and Cav-1 expression was analyzed by western blotting. Columns are means ±SD (*n = *5). *P<0.05 vs. *time 0. B:* Detached H460 cells were subjected to a monolayer culture of HUV-EC-C. The interaction of H460 and HUV-EC-C cells was examined in the presence of IL-1*β* (10 ng/ml). Phase-contrast microscopic pictures of representative experiments are shown. *C:* Correlation analysis of cellular Cav-1 level versus H460-HUV-EC-C adhesion (*n* = 5). *D:* Cav-1 overexpressed Hcav-1 or short hairpin Cav-1-transfected shCav-1 cells were constructed and analyzed for Cav-1 expression by Western blotting. Blots were re-probed with β-actin antibody to confirm equal loading of samples. The immunoblot signals were quantified by densitometry, and mean data from independent experiments were normalized to the results. Columns are means ±SD (*n = *3), *P<0.05 vs. H460 cells. *E:* Hcav-1, H460, and shCav-1 cells were detached for 3 h and added onto a culture of HUV-EC-C. Columns are means ±SD (*n = *3), *P<0.05 vs. H460 cells.

Cav-1 was shown to associate with metastatic potentials of lung cancer cells [6], [12], while its role on cancer cell adhesion is still unclear. To study the role of Cav-1 protein on cancer cell adhesion, we first detached the cancer cells to mimic the early step of metastasis, and evaluated the expression profile of Cav-1 after cell detachment over times. Lung cancer H460 cells cultured in ultralow attachment plates were analyzed for Cav-1 protein expression by western blotting at indicated time points. Figure 1A shows that after cell detachment, Cav-1 gradually decreased in a time-dependent fashion and the significant decrease was detected as early as 6 h after cell detachment. Next, the suspended cells at concurrent time points were subjected to the monolayer cell adhesion assay as described in *Materials and Methods*. Figures 1A and B show that after cell detachment, Cav-1 expression gradually decreased over time concomitant with the increase of cancer cell adhesion on endothelial surfaces, suggesting the potential role of Cav-1 in cancer adhesive regulation. We further supported such a correlation, by generating a correlation plot between Cav-1 expression and cancer cell adhesion (Fig. 1 C). Not only did the reduction of these proteins correlate well with the enhanced ability of cancer cells to adhere on endothelium surfaces, but the plot also revealed a highly correlated profile with the correlation coefficient of 0.922.

To confirm that Cav-1 attenuates cancer cell adhesion to endothelial cells, we explored the effect of different ectopic Cav-1 expression levels on H460 cells adhesion to endothelium. We established Cav-1 overexpressing (Hcav-1) cells, short-hairpin (sh)-mediated Cav-1 knockdown (shCav-1) cells by stable transfection. The transfectant clones were analyzed for Cav-1 expression compared to their parental H460 cells by western blotting (Fig. 1D). It is noteworthy that the control transfected cells generated from control plasmids, pDS_XB-YFP and control shRNA plasmid A, were also evaluated for Cav-1 expression, however; the level of the protein was comparable to that of H460 cells (data not shown). Cav-1 overexpressed, Cav-1 knockdown, and H460 cells were detached and subjected to endothelial adhesion assay and the cancer cells adhering on the HUV-EC-C endothelial cells were observed. As expected, analysis of cell adhesion showed that Cav-1 overexpressed cells exhibited the lowest ability to adhere to the HUV-EC-C cells, while the shCav-1 cells had the highest ability. Also, the vector transfected cells were similarly evaluated for adhesive capability and we found no significant alteration in comparison to that of parental cells (data not shown). This indicates that Cav-1 negatively regulates cancer cell adhesion to vascular endothelium.

### Caveolin-1 Suppresses Hydrogen Peroxide and Hydroxyl Radical Generation after Cell Detachment

**Figure 2:**
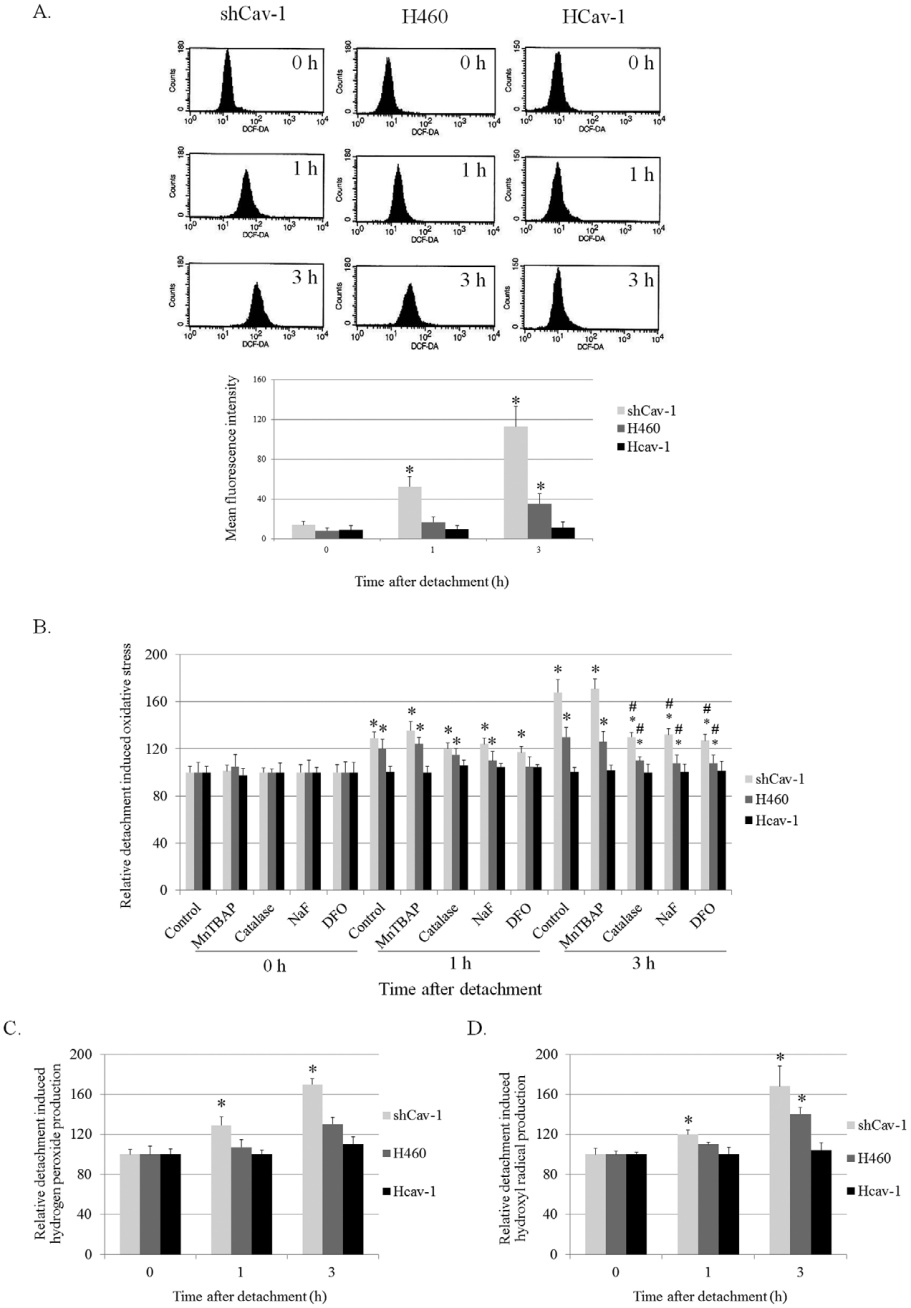
Caveolin-1 suppressed cell detachment-induced ROS generation. *A:* Cellular ROS level of detached H460, shCav-1, and Hcav-1 cells was determined by flow cytometry using H_2_DCF-DA as a fluorescence probe. Columns are means ±SD (*n = *3), *P<0.05 vs. *time 0. B:* H460, shCav-1, and Hcav-1 cells were treated with Mn(III)tetrakis(4-benzoic acid) porphyrin chloride (MnTBAP, 50 µM), Catalase (Cat, 7,500 U/ml), Sodium formate (NaF, 2.5 mM), or Deferoxamine (DFO, 1 mM). The intracellular ROS level of these cells was determined by H_2_DCF-DA probe. Columns are means ±SD (*n = *5), *P<0.05 vs. attached control (*time 0*), and #*P*<0.05 vs. non-treated control at corresponding time. *C:* Hydrogen peroxide level of the cells was determined by microplate reader using Amplex Red. Columns are means ±SD (*n = *3), *P<0.05 vs. control cells at *time 0. D:* Hydroxyl radical induction was determined by HPF. Columns are means ±SD (*n = *3), *P<0.05 vs. control cells at *time 0.*

Our previous study has shown that in the adhered condition, Cav-1 function in attenuating oxidative stress caused by hydrogen peroxide treatment [13]. Therefore, it is possible that Cav-1 protein may function to regulate redox status of the cancer cells during metastasis. To study the effect of Cav-1 protein on detachment-induced ROS generation, Cav-1 overexpressed (Hcav-1), Cav-1 knock-down (shCav-1), and control cells (H460) were detached and incubated in adhesion-resistant plates until specific time points. The suspended cells were then analyzed for intracellular ROS levels by flow cytometry using DCFH_2_-DA as a fluorescent probe. Figure 2A shows that cell detachment induced an increase in cellular ROS in a time-dependent manner which aligned with our previous report [6]. The present study revealed for the first time that Cav-1 functioned in attenuating the detachment induced ROS in these cells. ShCav-1 cells possessing the lowest Cav-1 expression was shown to exhibit the highest ROS induction and such an induction could be detected as early as 1 h after detachment (Fig. 2A), whereas cellular ROS in Cav-1 overexpressed (Hcav-1) cells was not altered in response to cell detachment.

Further, the specific ROS inhibitors were used to evaluate specific ROS which were up-regulated in these cells. Cav-1 overexpressed, Cav-1 knock down, and H460 cells were pretreated with specific ROS inhibitors which were MnTBAP (a superoxide anion inhibitor), catalase (a hydrogen peroxide inhibitor), deferoxamine (a hydroxyl radical inhibitor), and sodium formate (a hydroxyl radical scavenger), and the ROS levels after detachment for 0–3 h were determined. Figure 2B indicates that treatment with catalase, sodium formate, and deferoxamine could block the induction of ROS in these cells, suggesting that hydrogen peroxide and hydroxyl radical were primary specific ROS up-regulated after cell detachment.

The effect of Cav-1 on hydrogen peroxide and hydroxyl radical produced by these cells was next evaluated. Amplex red, a specific detection probe for hydrogen peroxide, and HPF, a specific detection probe for hydroxyl radical, were used to determine such specific ROS in the detached cells. Figures 2C and D indicate that cell detachment increased the signals of hydrogen peroxide and hydroxyl radical specific probes in a time-dependent manner. Importantly, the signal of either hydrogen peroxide or hydroxyl radical was barely detectable in Cav-1 overexpressed cells, whereas such ROS signals were potentiated in the cells expressing a low level of Cav-1. These results indicated that Cav-1 attenuated hydrogen peroxide and hydroxyl radical generations in suspended lung cancer cells.

### Hydrogen Peroxide and Hydroxyl Radical Regulate Cancer Cell Adhesion to Endothelial Cells via VCAM-1-dependent Mechanism

**Figure 3:**
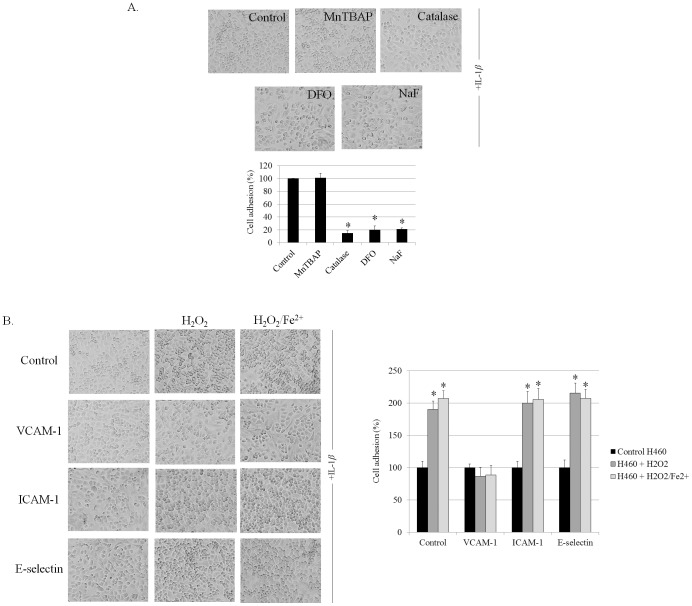
Hydrogen peroxide and hydroxyl radical enhance H460 cell adhesion to HUV-EC-C via VCAM-1. *A:* H460 cells were left untreated or pretreated with Mn(III)tetrakis(4-benzoic acid) porphyrin chloride (MnTBAP, 50 µM), Catalase (Cat, 7,500 U/ml), Sodium formate (NaF, 2.5 mM), or Deferoxamine (DFO, 1 mM) then detached for 3 h prior to adhesion assay. Columns are means ±SD (*n = *3), *P<0.05 vs. non-treated control. *B:* H460 cells were treated with 100 µM H_2_O_2_ or 100 µM H_2_O_2_ and 50 µM FeSO_4_ and subjected to the HUV-EC-C surface which was blocked by VCAM-1, ICAM-1, or E-selectin antibodies. Phase-contrast microscopic pictures of representative experiments are shown. Columns are means ±SD (*n = *3), *P<0.05 vs. non-treated control.

ROS were shown in many studies to play a key role in the regulation of cancer cell behaviors [11]. Together with the above finding indicating that Cav-1 attenuated hydrogen peroxide and hydroxyl radical formations during cell detachment, we investigated whether hydrogen peroxide and hydroxyl radical play a role in regulating cancer cell adhesion. Lung cancer cells were incubated with various known specific ROS modulating agents as described and the cells were subjected to the adhesion assays. Figure 3A indicates that treatment of the cells with a superoxide inhibitor MnTBAP had only an minimal and insignificant effect on cancer cell adhesive activity, whereas the addition of catalase, deferoxamine, and sodium formate significantly inhibited the adhesion of H460 cells to endothelium surface. These results suggested that hydrogen peroxide and hydroxyl radical but not superoxide anion are the ROS that potentiate adhesive capability of these cancer cells to vascular endothelial cells.

Evidences have suggested that endothelial cell adhesion molecules (ECAMs) play an important role on cancer-endothelial cell interaction [15]. Activation of endothelial cells by IL-1*β* induced the expression of ECAMs namely VCAM-1, ICAM-1 and E-selectin on endothelial cell surfaces, and these proteins facilitated the binding of cancer cells [15]. To confirm the role of hydrogen peroxide and hydroxyl radical on cancer-endothelial cell interaction and to define the affected adhesion molecules, we blocked the endothelial surfaces with specific antibodies against VCAM-1, ICAM-1 and E-selectin, prior to cancer cell adhesion. Also, the cancer cells were treated with exogenous hydrogen peroxide in the absence or presence of ferrous sulfate to confirm the role of hydrogen peroxide and hydroxyl radical, respectively.

Figure 3B indicates that hydrogen peroxide and hydroxyl radical significantly enhanced cancer cell adhesion to endothelial cells, confirming the role of mentioned ROS on cancer cell adhesion. Interestingly, blocking endothelium with the VCAM-1 antibodies could abolish the effect of hydrogen peroxide and ferrous sulfate on the adhesive property of these cancer cells while antibodies against ICAM-1 and E-selectin had no significant effects. These results indicated that hydrogen peroxide and hydroxyl radical may, at least in part, regulate the interaction of the cancer cells to endothelial surface through VCAM-1-dependent mechanism. Also, Cav-1 may decrease the adhesive property of the cancer cells by attenuating hydrogen peroxide and hydroxyl radical.

### Cav-1 Suppresses ROS Generation after Cell Detachment through PI3K/Akt Pathway

**Figure 4:**
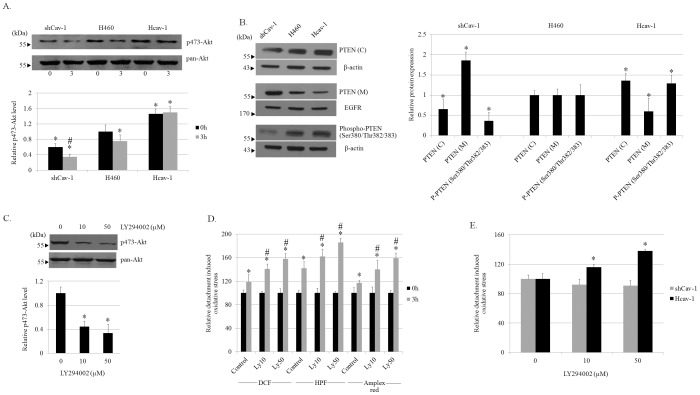
Caveolin-1 suppresses hydrogen peroxide and hydroxyl radical inductions through PI3K/Akt pathway. *A:* H460, shCav-1, and Hcav-1 cells were detached and suspended in poly-HEMA-coated plates for 0–3 h. p473-Akt and pan-Akt level in these cells was determined by antibodies specific to p473-Akt and pan-Akt. Columns are means ±SD (*n = *3), *P<0.05 vs. H460 cells at *time 0*, *^#^P*<0.05 vs. corresponding control cells at *time 0*. *B:* Lysate of H460, shCav-1, and Hcav-1 cells were used for the investigation of PTEN, phospho-PTEN (Ser380/Thr382/383) expression. Caveolae microsomal fraction (M) was prepared as described in materials and methods and used for the investigation of PTEN (M). EGFR was used as a marker for the caveolae fraction. Columns are means ±SD (n = 3), *P<0.05 vs. H460 cells. *C:* H460 cells were left untreated or pretreated for 2 h with 10 and 50 µM LY294002 then the cells were detached for 3 h and analyzed for p473-Akt and Akt levels. Columns are means ±SD (*n = *3), *P<0.05 vs. non-treated control. *D:* H460 cells were treated with LY294002 and determined for ROS, hydroxyl radical, and hydrogen peroxide production by DCFH_2_-DA, HPF, Amplex Red, respectively. Columns are means ±SD (*n = *4), *P<0.05 vs. non-treated control at *time 0*, #*P*<0.05 vs. non-treated control at *3 h. E:* ShCav-1 and Hcav-1 cells were treated with LY294002 and analyses for cellular ROS using DCFH_2_-DA. Columns are means ±SD (*n = *3), *P<0.05 vs. non-treated control.

Having shown that Cav-1 attenuated cancer ability to adhere to an endothelium by decreasing cellular hydrogen peroxide and hydroxyl radical up-regulations, and evidence indicated that Cav-1 regulates several cell behaviors during cell detachment through PI3K/Akt pathway [11]. To further confirm the role of Cav-1 on Akt signaling, we monitored the Akt activation in terms of phosphorylated (serine 473) Akt. In agreement with previous finding [16], we found the increased level of Akt activation in Hcav-1 cells in comparison to that of H460 and shCav-1 cells (Fig. 4A). Also, we found that cell detachment significantly decreased the level of activated Akt in almost all cells. While phosphorylated (serine 473) Akt in shCav-1 cells found to be most affected by such a cell detachment, the level of activated Akt in Hcav-1 was barely altered (Fig. 4A).

Since phosphatase and tensin homolog deleted on chromosome ten (PTEN) is a major negative regulator of the PI3K/Akt signaling pathway, we also performed the experiment to elucidate the role of Cav-1 on PTEN. Our results indicated that PTEN was up-regulated in the cytoplasmic portion (C) but down-regulated in the caveolae microsomal fraction (M) in Cav-1 overexpressed (Hcav-1) cells in comparison with those of H460 cells (Fig. 4B). In contrast, Cav-1-knock down cells exhibited the opposite profiles, suggesting that Cav-1 regulated the compartmentalization of PTEN. In order to assess the activity of PTEN, the western blot analysis of the C-terminal phosphorylated PTEN at Ser380, Thr382, and Thr383 that promote PTEN stability but decrease PTEN activities [17], was performed. Figure 4B shows that activated PTEN was significantly reduced in the Hcav-1 cells with the observations that the phosphorylated PTEN (at Ser380, Thr382, and Thr383) were significantly increased. Together with the finding indicating that the phosphorylation of such a protein in ShCav-1 cells was decreased, Cav-1 was demonstrated in the presence study that negatively regulated activated status of PTEN and such regulation may, at least in part, sustained the level of phosphorylated Akt.

To test whether the alteration in phosphorylated Akt affects the cellular oxidative stress, a PI3K inhibitor LY294002 was used to suppress Akt activation. H460 cells were incubated with PI3K inhibitor LY294002 at the concentrations of 10 and 50 µM, and Akt and phosphorylated-Akt levels were determined by western blotting (Fig. 4C). LY294002 at 10 and 50 µM were able to reduce p473-Akt (Fig. 4C). The effect of LY294002 on ROS induced by cell detachment was monitored by DCFH_2_-DA, HPF and Amplex red. Apparently, PI3K inhibitor was able to increase cellular oxidative stress as indicated by the significant increase of DCF fluorescence signal. Moreover, the specific ROS, namely hydroxyl radical and hydrogen peroxide, were found to be significantly increased in response to the treatment of PI3K inhibitor in H460 cells (Fig. 4D).

Interestingly, the treatment of LY294002 at 10 and 50 µM could reverse to effect of Cav-1 in suppressing ROS reduction in Hcav-1 cells. Figure 4E shows that ROS signal in Hcav-1 cells treated with LY294002 was remarkably increased, compared to that of non-treated Hcav-1 cells. In contrast, ROS level in shCav-1 cells were barely affected by the treatment of LY294002. Taken together, these findings suggested that Cav-1 regulate ROS formation after cell detachment by sustaining the activation of PI3K/Akt pathway.

## Discussion

So far, a number of cancer-related proteins have been identified and used for specific applications, including biomarkers for the early detection, prognosis, and molecular targets for anti-cancer design [18]. According to the widely accepted hypothesis that not all primary tumors are able to adhere on the endothelial surfaces, but certain cancer cells possessing an innate or adaptive ability are able to adhere on the surface of vascular endothelial cells and to form metastases [19]. Many regulatory proteins were identified as tumor suppressor or tumor promoter proteins; however, in the case of Cav-1, both functions were reported. Cav-1 was first described as a tumor suppressor protein since the down-regulation of Cav-1 was found during cell transformation [7]. In contrast, Cav-1 was shown to potentiate cancer progression and increasing evidence has indicated its role as a cancer promoter [20]. Cav-1 was shown to function as an important mediator for multiple pro-survival signaling pathways [21]–[23]. In addition, Cav-1 expression was shown to increase in several types of human cancers, including lung, breast, prostate, and pancreas cancers, and this up-regulation was associated with a high degree of metastasis [20], [24]–[28]. Even though Cav-1 was shown to potentiate lung cancer metastasis by enhancing anoikis resistance [9] and invasion and migration [11], the present study provided the inhibitory effect of Cav-1 on cancer-endothelium adhesion which has not been demonstrated.

Hydrogen peroxide and hydroxyl radical previously shown to be generated during cell detachment [6] were demonstrated herein to be critical for adhesive action of cancer cells to endothelial surfaces (Fig. 3B). Such a finding has strengthened the role of ROS, especially hydroxyl radical in the regulation of cancer-endothelial interaction since hydroxyl radical was reported to enhance cancer cell adhesion by affecting the surface of endothelial cells [29]. Our finding was added to the growing list of the functions of specific ROS in regulating cellular pathologic events.

Cell adhesion molecules (CAMs), such as vascular endothelial cell adhesion molecule-1 (VCAM-1), intercellular cell adhesion molecule-1 (ICAM-1), and E-selectin, have been shown to be essential for endothelial cells in interaction with the cancer cells [15]. Such CAMs were shown to be up-regulated when endothelial cells were exposed to inflammatory cytokines, namely interleukin-1 beta (IL-1β) and tumor necrosis factor-alpha (TNF-α) [15]. Among them, VCAM-1 has garnered increasing attention in the anti-cancer field and recognized as a potential therapeutic target in metastasis [30]. In the present study, the regulatory role of hydrogen peroxide and hydroxyl radical was found to be associated with the expression of VCAM-1 on the endothelial cells (Fig. 3B). Blocking VCAM-1 on the endothelium surface but not ICAM-1 and E-selectin was shown to abolish the effect of such ROS on cancer cell binding, implying that ROS mediates cancer cell adhesion via VCAM-1-dependent mechanism. Since VCAM-1 is an endothelial ligand for interacting with β1 sub family of integrins [31], we hypothesized that hydrogen peroxide and hydroxyl radical probably affected the expression level and/or function of integrins on cancer cell surfaces.

**Figure 5:**
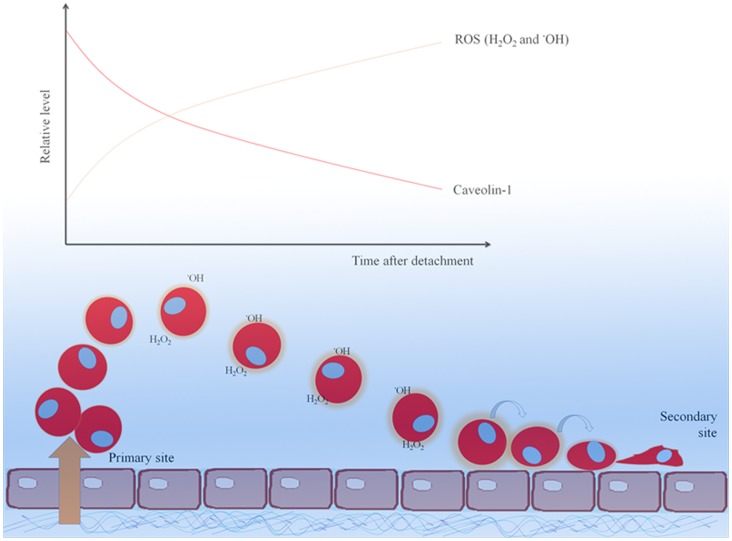
The scheme represents the dynamic alterations of cellular Cav-1 and ROS levels and their possible effect on cancer cell adhesion to vascular endothelium. The present study reveals that Cav-1 inhibits cancer-endothelium interaction. During detachment, the decrease of Cav-1 resulted in the increase of hydrogen peroxide and hydroxyl radical along with enhanced cancer-endothelium adhesion.

The down-regulation of Cav-1 in lung cancer cells was shown to be dependent on the cellular redox status [6]. However, the feedback mechanism of Cav-1 on ROS regulation has not been reported elsewhere. We found that Cav-1 suppressed the formation of ROS in response to cell detachment and the inhibition of hydrogen peroxide and hydroxyl radical was essential for anti-adhesive role of Cav-1. The ectopic overexpression of Cav-1 significantly attenuated ROS, including hydrogen peroxide and hydroxyl radical generations induced by cell detachment, while the transfection with shRNA mediating Cav-1 down-regulation showed the opposite effect (Fig. 2A–D). Further, we showed that Cav-1 plays a role in regulating ROS after cell detachment through PI3K/Akt pathway. Considering the fact that Cav-1 was shown to reduce overtimes after cell detachment and this reduction was associated with the increase of cancer cell adhesion (Fig. 1B), at later time the metastatic cells may adhere and form secondary tumors. Also, Cav-1 was shown to reduce anoikis response that allows cancer cells expressing high Cav-1 to survive for long time and adhered to the distant sites (Fig. 5).

Although activated status of Akt was shown to be regulated by several mechanisms, several cancer-related studies suggested that PTEN play a dominant role in attenuating Akt functions [32]–[33]. Indeed, phosphatase activity of PTEN turns phosphatidyl-inositol 3,4,5 triphosphate (PIP_3_), an activator of Akt, into PIP_2_, resulting in Akt suppression [32]. In the present study, PTEN expression was found in the caveolin-1 rich domains and was down-regulated in such a cell compartment in the Cav-1-overexpressed cells (Fig. 4B). In contrast, the level of PTEN was found to be increased in cytoplasmic portion of the cells. These results suggested that both Cav-1 and PTEN shared the same localization in these cells. In terms of PTEN function, we found that phosphorylated PTEN was significantly up-regulated in Cav-1-overexpressed cells, while the Cav-1-knock down cells exhibited the opposite results (Fig. 4B) Although further investigations on the underlying mechanisms that Cav-1 regulates PTEN are needed, our findings indicate that Cav-1 sustained the activity of Akt by suppressing PTEN activation.

In summary, the present study has revealed a novel finding on the cancer-endothelium regulatory effect of Cav-1 in human lung carcinoma H460 cells. This effect of Cav-1 involves its ability to suppress hydrogen peroxide and hydroxyl radical generation by sustaining phosphorylated Akt levels. Because cancer adhesion to endothelial cells is crucial for cancer metastasis, the findings of this study could be beneficial to the understating of cancer metastasis and development of cancer therapy.
